# What is the most dangerous sexual position that caused the penile fracture? A systematic review and meta-analysis

**DOI:** 10.1590/S1677-5538.IBJU.2023.0419

**Published:** 2024-03-18

**Authors:** Syarif Syarif, Abdul Azis, Ahmad Shafwan Natsir, Muhammad Zulharyahya Dandy Asmara Putra

**Affiliations:** 1 Hasanuddin University Faculty of Medicine Makassar South Sulawesi Indonesia Faculty of Medicine, Hasanuddin University, Makassar, South Sulawesi, Indonesia; 2 Mulawarman University Faculty of Medicine Samarinda East Kalimantan Indonesia Faculty of Medicine, Mulawarman University, Samarinda, East Kalimantan, Indonesia

**Keywords:** Penis, Men's Health, Systematic Reviews as Topic

## Abstract

**Purpose:**

Penile fracture (PF) affects 1,14 to 10,48 men in every 100.000 men in East Asia, and the primary aetiology is sexual intercourse, but the knowledge regarding the most dangerous sexual position is not well explained. This study compares three sexual positions: man on top position (MTP), woman on top position (WTP), and doggy style position (DSP), leading to PF potential.

**Materials and methods:**

A search of sexual position-related PF in Google Scholar, PubMed, Cochrane, and PMC Europe was performed. Criteria inclusion was the full text of relevant articles which describ the number of sexual positions. It was analyzed by odds ratio, random model effect, and the OR and 95%CI were calculated.

**Results:**

Twelve relevant papers involving 490 patients comprised 169 MTP, 120 WTP, 158 DSP, and 43 no intercourse cases. Meta-analysis of all sexual positions was a MTP P= 0,04, WTP P=0,49, and DSP P=0,0005.

**Conclusions:**

The man-dominant positions (MTP and DSP) were significantly potential for PF, which speculated that when a man is dominant and very excited, intercourse may become highly vigorous and impact trauma. This study found that man's dominant position consists of DSP and the MTP significantly lead to PF.

## INTRODUCTION

Sexual intercourse is a routine activity with various positions involved. In men, the rudimental pressure on the penis can sometimes lead to a condition called penile fracture, which is a rare injury. Penile fracture (PF) occurs in about one out of every 175,000 men in the United States and 1,14 to 10,48 out of every 100,000 men in East Asia (1). The increase in the population has led to a rise in the number of such injuries (2). Because mechanical factors typically cause this injury, it is possible to take steps to prevent it.

The mechanism of penile fracture (PF) initiates when the tunica albuginea of the corpora cavernosum ruptures as a result of a traumatic process. The classic triad of symptoms, including a distinctive “cracking” sound, immediate detumescence (loss of erection), and intense pain, serves as valuable indicator for diagnosing PF. The injury can progress to include hemorrhage, leading to urethral bleeding and difficulties with urination (3). Without appropriate treatment, penile fracture (PF) can progress to various complications, including erectile dysfunction, curvature of the penis, and the formation of nodules (4).

Sexual intercourse is the primary aetiology of PF (46% of cases), while the other causes include masturbation and forced bending of the penis (4). Previous research revealed variations in the number of sexual positions associated with PF. When defining sexual position terminology, there is significant diversity, but categorization will be done into three positions based on dominance: Woman on Top Positions (WTP), Man on Top Positions (MTP), and Doggy Style Positions (DSP). WTP represents a position where the woman is on top, assuming a dominant role in the sexual relationship. MTP and DSP are positions where the man assumes a dominant role in sexual activity, with differences in how couples face each other in both positions. These positions can also be performed in various conditions, including lying, sitting, and standing (5).

The treatment of PF has evolved with several surgical techniques in use today, including distal circumcision-degloving or vertical penoscrotal techniques, with the former being the most popular. The advantage of the subcoronal degloving incision technique is that it allows for a full corporal body inspection, aids in detecting contralateral corporal body or urethral injuries, and facilitates easier repairs with natural cosmetic results. Some techniques combine both methods, with the penoscrotal incision as an additional option (6).

Understanding the potential risk associated with specific sexual positions is crucial for promoting sexual health and well-being and preventing penile fractures. In this comprehensive study, the authors conduct a systematic review and meta-analysis of global research to analyze the relative risks posed by three common sexual positions: man on top (MTP), woman on top (WTP), and doggy style position (DSP). By offering evidence-based insights and fostering awareness, this study aims to reduce the occurrence of penile fractures, enhance sexual well-being, and ensure timely and appropriate management when needed.

## MATERIALS AND METHODS

### Study design and search strategy

In May-June 2023, a systematic review and meta-analysis were conducted with the following PRISMA-P 2015 checklists to investigate the association between various sexual positions (including MTP, WTP, and DSP) and the risk of PF. It was collected for odds ratios (OR) and 95% confidence interval (95%CI) using a random effect model. The electronic source was browsed in Google Scholar, PubMed, Cochrane, and PMC Europe until May 2023. The key words included “Sexual habit” or “Sexual position” or “Woman on top” or “Women on top” or “Andromache” or “Partner on top” or “Man on top” or “Men on top” or “Missionary” or “Partner below” or “Doggy style” or “Behind partner” and “Penile Fracture” or “Penile rupture”. The literature sources were limited to full-text articles, open-access journals, English publications, and analytical research. The full text was cross-checked to exclude the missing items.

### Eligibility Criteria and Quality Assessment

The retrospective, prospective, cross-sectional, preprint, and case series studies with three or more cases of PF and information on any sexual position were included. Studies related to (1) autoerotism (masturbation), (2) unclear positions explanation, (3) unrelated titles and abstract, (4) reviews and commentary, and (3) double publication were excluded. Three independent authors assessed each paper's quality using the Newcastle-Ottawa Scale (NOS). The NOS assigned a score from 0 to 9, divided into three categories: selection (1-4), comparability (1-2), and outcome (1-3) with interpretations of reasonable (7-9), moderate (5-6), and poor (0-4). Discrepancies among authors were resolved through consensus.

### Data extraction and Outcome measure

Data extraction from each study contained (1) first author name, (2) publication year, (3) country of origin, (3) mechanisms of trauma, (4) sample size of each sexual position, and other relevant information. Three independent authors ensured the accuracy of data extraction. A comprehensive analysis compared all these sexual positions: partner below/MTP vs. others, partner on top/WTP vs. others, and behind partner/DSP vs. others.

### Statistical Analysis

Meta-analysis was conducted using Review Manager 5.4. The Mantel-Haenszel formula with random-effect models was employed to calculate OR and 95%CI for the PF outcome, regardless of heterogeneity. Heterogeneity was assessed using I-squared statistic (I2), with interpretations of <25% indicating a low degree, 26-50% a moderate degree, and >50% a high degree. Funnel plot analysis was performed to assess the qualitative risk of publication bias.

## RESULTS

### Study Selection

**Figure 1:**
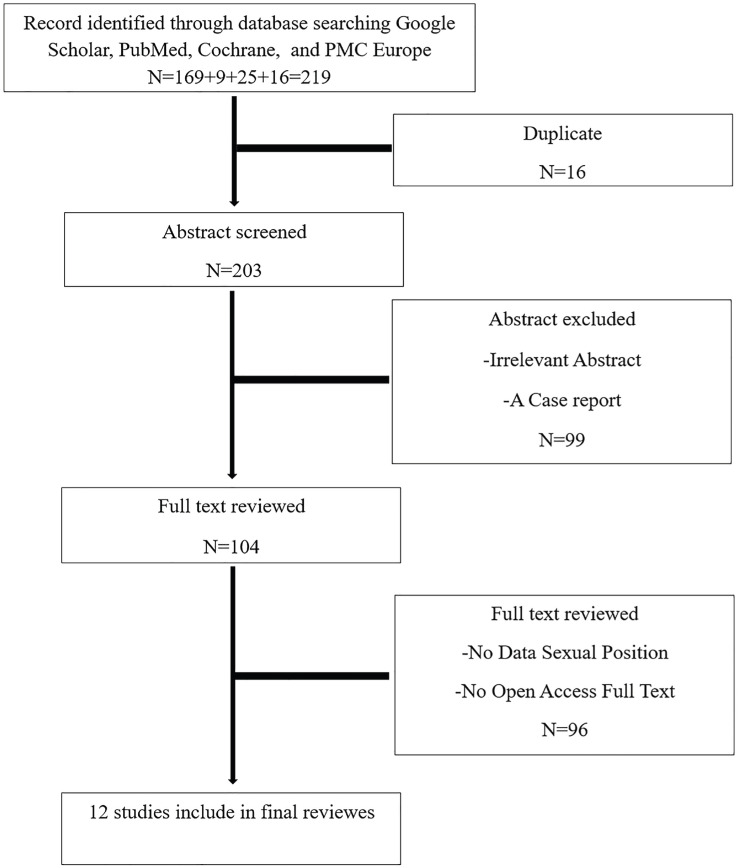
Eligibility pathway. A total of 12 papers were included for the final systematic review and meta-analysis.

A total of 12 studies suitable for inclusion in the meta-analysis were identified through the final search strategy (Table-1). Initially, 219 studies were found, but 16 papers were excluded due to duplication, 99 papers were excluded because they pertained to irrelevant topics or constituted a single case report, and 96 papers were excluded due to a lack of registered data on sexual position and not provided open access to the full text (Figure-1).

**Table 1:** List of Studies. Following the application of eligibility criteria, a total of 12 papers were incorporated into the study. These papers encompassed a total of 490 cases of PF, which were compared across various sexual positions, including WTP, MTP, and DSP. Note: WTP (Woman on Top Position); MTP (Man on Top Position); and DSP (Doggy Style Position).

Study	Country	Sample	MTP	WTP	DSP	Information of study Conclusion
Barros et al., 2020 (7)	Brazil	255	103	31	110	MTP and DSP have more associations with bilateral PF of the corpus cavernosum and urethral lesions.
Can et al., 2021 (8)	Turkey	16	7	9	0	Ejaculation time was longer after PF
TAC et al., 2021 (9)	Marocco	47	11	14	22	It's essential to act quickly by avoiding specific sexual postures (DSP), getting timely surgery, and taking a break from sexual activity to protect from PF.
Ghous et al., 2021 (10)	Pakistan	18	12	0	0	MTP was the most precarious position observed
Magaña-González et al., 2019 (11)	Mexico	25	4	12	9	Getting surgery quickly after diagnosis, usually within 35 hours, leads to better outcomes.
Mensah et al., 2010 (12)	Ghana	3	2	1	0	PF is diagnosed clinically, and prompt surgical repair fully restores sexual function.
Mir et al., 2017 (13)	India	26	20	0	6	Proper history and clinical examination easily reach PF diagnosis and mode of trauma.
Nason et al., 2013 (14)	Ireland	20	0	13	0	In a small group of men with PF treated quickly and could still have erections, their long-term sexual satisfaction seems good.
Pavan et al., 2017 (15)	India	19	3	14	2	PF undergoing delayed repair has preservation of erectile potency, and overall sexual function is maintained.
Pavan et al., 2014 (16)	Italy	8	1	4	3	Psychologically, penile trauma intensifies the fear of reoccurrence but decreases with time.
Reis et al., 2014 (3)	Brazil	32	6	14	6	The riskiest sexual position was WTP, but getting surgery right away led to very few long-term problems.
Tijani et al., 2012 (2)	Nigeria	21	0	8	0	The reasons behind PF have changed in the author's area over the past two years, possibly due to population shifts.

**Figure 2:**
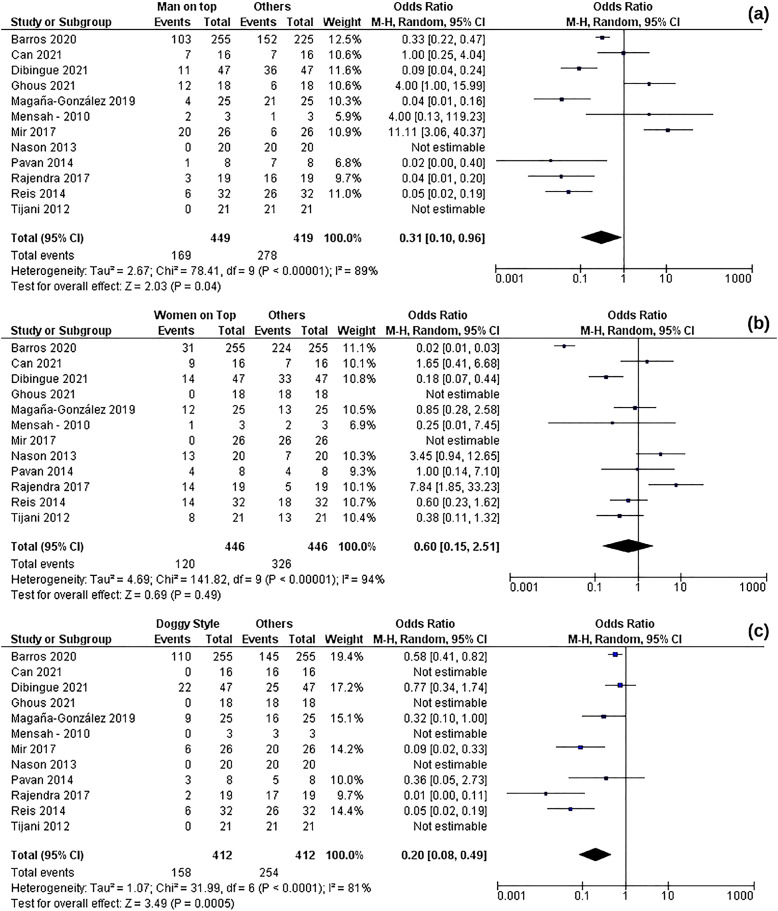
Comparison of Forest Plot for Three Sexual Positions. WTP (b) shows no significant relation to PF, while MTP (a) and DSP (c) exhibit a significant association with PF.

**Figure 3:**
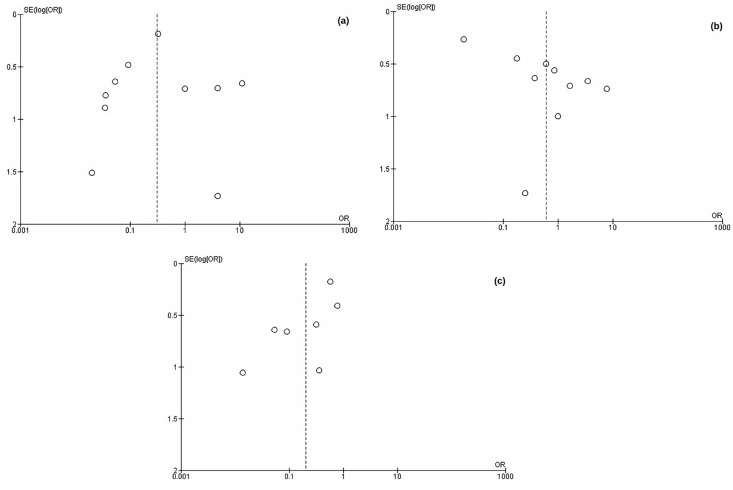
Comparison of Funnel Plots for Three Sexual Positions. All the funnel plots display asymmetry, indicating a potential publication bias.

### Data Synthesis and Publication Bias

A comparison was made among a total of 12 papers that examined three sexual positions: MTP in 10 studies, WTP in 10 studies, and DSP in 7 studies. These papers included 490 case studies, with 169 cases involving MTP, 120 involving WTP, 158 involving DSP, and 43 cases where no intercourse was reported. Separate analyses were conducted for each position. It is worth noting that three journals with similar themes and locations were published by Barros et al., and only the most recent one was included due to variations in sample sizes among the journals, resulting in asymmetric funnel plots in each subgroup.

#### 1 – Man on top position

A significant risk of penile fracture associated with the MTP position was indicated by analysis of this group (OR 0,31; 95% CL: 0,1-0,96; P= 0,04). However, it should be noted that there is a high degree of heterogeneity (I2= 89%) within the studies related to this position. An analysis of the funnel plot for the risk of PF in the man-on-top position revealed an asymmetric plot, suggesting potential publication bias.

#### 2 – Woman on top position

In the case of WTP, a non-significant estimate was observed for the risk of PF (OR 0,60; 95% CL: 0,15-2,51; P=0,49). Similar to the man on top, there is considerable heterogeneity (I2= 94%) within the studies about this position. The funnel plot analysis for the risk of PF in the WTP also indicated an asymmetric hinting at potential publication bias.

#### 3 - Doggy Style position

A significant estimate for the risk of PF associated with the DSP was found (OR 0,20; 95% CL: 0,08-0,49; P= 0,0005). Heterogeneity in this position was also high (I2= 81%), and once again, the asymmetric funnel plot potential publication bias.

All positions exhibited high heterogeneity, but this was expected due to the random effect model employed. When the P-value was across all positions, it became apparent that doggy style presented the most significant risk of PF, indicating that it was the most likely to lead to this injury.

## DISCUSSION

PF is considered a critical medical emergency, typically resulting from basic rudimental mechanisms, with sexual intercourse being the most common cause (4). In this study, 12 papers were identified and analyzed to compare three sexual positions potentially leading to PF. Interestingly, the DSP and MTP have not received attention in the existing literature. There is a hypothesis that in cases where a man assumes the dominant position and is highly aroused, sexual intercourse becomes vigorous, potentially leading to trauma when the penis accidentally slips out before entering the vagina (17). Both positions have shown more associations with bilateral fractures of the corpus cavernosum and urethral injuries (7).

While sexual intercourse is identified as the primary aetiology of PF along with autoerotism and penile manipulation (7,10), it is worth noting that, despite men predominantly assuming dominant positions in sexual enter counters, WTP was a major focus in seven studies within this review. It should not downplay the potential and risks associated with this position. However, the limited sample size of WTP studies may not be sufficient to counterbalance the significant association of the dominant male position. Furthermore, the body mass index of the sexual partner in WTP may also contribute to PF (8).

Symptoms and treatment specific to each sexual position were not delineated in the studies. Generally, PF is characterized by a bent penis with detumescence and intense pain. Can et al. explain that penile fracture contributes to a longer ejaculation time (8). Immediate treatment is crucial, though fear, embarrassment, and delaying referral can sometimes result in postponed surgery (15). All papers explain that immediate surgery provides a better result and esthetic of the penile, which must be done before 35 hours (11). It also enhances the patient's confidence and sexual function after the surgery (12,16). The standard repair method involves a procedure known as degloving (4,13).

As mentioned earlier, the enthusiastic sexual intercourse associated with a man's dominant position can increase the risk of PF. It underscores the importance of men being mindful and exercising caution during intercourse. This concern is directly related to controlling the dynamic of intercourse and warrants further research into a regulation method.

This study's extensive inclusion of research from diverse global regions and data from various cultural backgrounds strengthens the validity and generalizability of our findings. It broadens our understanding of PF and associated risk factors across different populations. By incorporating this rich dataset, our research provides a globally relevant perspective on sexual health and penile fracture prevention.

Nevertheless, this research has limitations that must be addressed in future studies. The other factors that can influence PF, such as age, penile size, and anatomical abnormalities of the penis, should be compared with the sexual positions in future studies. Our findings offer valuable educational insights for prevention. As alluded to above, the scientific cause for why the man's dominant positions contribute more than WTP remains one of the study's limitations. Future research is needed to evaluate the traumatic potential injury of both positions. Another limitation is the relatively small sample size in all position categories, and most of the included papers had a cross-sectional design. Further research is needed to address these limitations and provide a more comprehensive understanding of the relationship between sexual positions and PF.

## CONCLUSION

This study explores the sexual position that may contribute to PF and concludes that the man's dominant positions, including DSP and MTP, significantly increase PF risk. In contrast, the WTP does not significantly impact PF. For more comprehensive results, future research should consider additional factors such as age, penile size, and anatomical abnormalities of the penis.
